# Spatiotemporal variations in dissolved organic carbon in China’s major river basins and their associations with climate change and human activities

**DOI:** 10.1186/s13021-025-00387-0

**Published:** 2025-12-27

**Authors:** Yanru Sun, Anzhi Wang, Lidu Shen, Yage Liu, Yuan Zhang, Rongrong Cai, Wenli Fei, Jiabing Wu

**Affiliations:** 1 CAS Key Laboratory of Forest Ecology and Silviculture, Institute of Applied Ecology, Chinese Academy of Sciences, Shenyang, 110016 China; 2 University of Chinese Academy of Sciences, Beijing, 100049 China

**Keywords:** Dissolved organic carbon, Spatiotemporal variations, Climatic factors, Human activities, Major rivers, China

## Abstract

Riverine dissolved organic carbon (DOC) is a vital element of regional carbon cycling, yet its magnitude and influencing factors remain poorly quantified. Existing large uncertainties in the distribution, trends, and drivers of DOC compromise the accuracy of terrestrial carbon budget estimations. This study compiled 1922 DOC data points from literature on four major Chinese river basins (i.e., the Songhua River Basin, Yellow River Basin, Yangtze River Basin, and Pearl River Basin) for the period 1997–2023. The spatiotemporal patterns and driving mechanisms of DOC in these basins were quantified and systematically analyzed. Key results are as follows: [1] Spatially, DOC concentration (C_DOC_) exhibited a distinct “north high, south low” pattern nationally, while DOC flux (F_DOC_) displayed an inverted “south high, north low” distribution. Temporally, C_DOC_ in the four basins all showed a statistically significant increasing trend, with an average annual rise of 0.04 mg L⁻¹ yr⁻¹. Meanwhile, the F_DOC_ into the sea in the Yangtze River Basin and Yellow River Basin also exhibited a statistically significant increase, with an average annual growth of 0.05 Tg yr⁻¹ [3]. Attribution analysis indicated that the spatiotemporal distribution of C_DOC_ was influenced by both climatic factors and human activities, whereas that of F_DOC_ was controlled primarily by streamflow. The findings of this study reflect the national distribution and dynamics of DOC in major Chinese rivers, and provide a valuable framework together with details of key parameters to support future research into global riverine carbon cycle models.

**Supplementary Information:**

The online version contains supplementary material available at 10.1186/s13021-025-00387-0.

## Introduction

Rivers serve as a critical carbon bridge between land and ocean. Annually, they transport approximately 260 Tg of dissolved organic carbon (DOC) to the oceans, with notable implications for the carbon cycle both regionally and globally [1–4]. However, quantitative studies on riverine DOC transport remain limited. The uncertainties in its spatiotemporal trends and driving mechanisms greatly hinder the accuracy of regional carbon budget assessments. Therefore, clarifying the spatiotemporal distribution characteristics of riverine DOC and its driving factors is of crucial scientific importance for improving regional carbon management strategies and optimizing global carbon cycle models.

Earlier studies have shown that significant changes have occurred in both DOC flux (F_DOC_) and DOC concentration (C_DOC_) in North America and Europe [5–10], as well as in other regions worldwide, such as the Congo River [11, 12], Lena River [13], and Thames River [14]. However, the underlying mechanisms behind these changes vary considerably. For example, in the Thames River Basin of the United Kingdom, C_DOC_ doubled during 1884–2014, with urbanization accounting for 90% of the long-term increase. During World War II, agricultural expansion (grasslands to croplands) caused a short-term peak, releasing 45 kt C [14]. Research in the United States found that from 1985 to 2018, F_DOC_ was controlled primarily by precipitation (positively correlated), while reduced sulfur deposition alleviated soil acidification, thereby promoting DOC release [15]. Long-term simulations have shown that climate change and rising CO₂ concentrations during 1901–2014 have driven a 33% increase in total carbon transport in Europe, with a 20% increase in export flux [10]. Observations in Arctic permafrost regions demonstrated that permafrost thawing and flood processes have greatly enhanced DOC release, with concentrations closely linked to active layer depth and soil temperature [13]. Such changes are attributed primarily to changes in air temperature and precipitation, as well as land use change and human activities.

As a key component of global land-ocean carbon flux, China’s rivers annually transport approximately 2.80–6.87 Tg of dissolved organic carbon (DOC) to the ocean [16, 17], accounting for 4.4%–22.5% of the total dissolved organic carbon flux from global rivers to the Pacific Ocean (30.50–64.13 Tg) [18–21]. Notably, F_DOC_ from four typical basins—the Songhua River Basin, Yellow River Basin, Yangtze River Basin, and Pearl River Basin—accounts for over 65% of China’s total F_DOC_ export to the ocean [22]. This proportion closely matches their combined contribution to the national total river streamflow (approximately 65%) [17], strongly suggesting that hydrological dominance (i.e., water flux) is the primary controlling factor of the F_DOC_ pattern at the national scale. These rivers span subtropical to cold temperate zones and flow through plateau permafrost regions, agricultural plains, and densely urbanized areas. The spatiotemporal variation in DOC levels reflects the impact of both natural geographic factors and anthropogenic influences on the carbon cycle. However, our systematic understanding of DOC dynamics in major Chinese rivers remains limited, highlighting critical gaps in regional carbon cycle research. Most previous related studies focused on individual river basins such as the Yangtze River Basin [23], the Yellow River Basin [24], or the Songhua River Basin [25], and lacked comparative and synthetic analyses across these representative basins.

Additionally, prior research relied mostly on limited intra-annual data [26, 27], with few long-term, multiyear quantitative analyses of DOC in representative Chinese basins. Although some recent studies have attempted to investigate the spatiotemporal patterns and influencing factors of DOC in China’s major rivers [28, 29], the reliability of their conclusions is generally hampered by insufficient data coverage both spatially and temporally.

To address the aforementioned issues, this study aims to investigate the spatiotemporal evolution of C_DOC_ and F_DOC_ in China’s four major river basins (the Songhua River, Yellow River, Yangtze River, and Pearl River), as well as to assess the relative contributions of climate change and human activities on these evolutions. To this end, C_DOC_ observation data from 1997 to 2023 were systematically compiled, and F_DOC_ was calculated by integrating runoff data. The temporal distribution trends of both C_DOC_ and F_DOC_ were then analyzed using the Mann–Kendall method. Additionally, the structural equation modeling (SEM) method was used to quantify the relative contributions of climatic factors (e.g., air temperature and precipitation) and human activity factors (e.g., land use change, population density, and gross domestic product (GDP)) to the spatiotemporal changes in C_DOC_ and F_DOC_. The findings offer scientific evidence for refining regional carbon management strategies, and identify key parameters for global riverine carbon cycle models.

## Materials and methods

### Study area

**Fig. 1:**
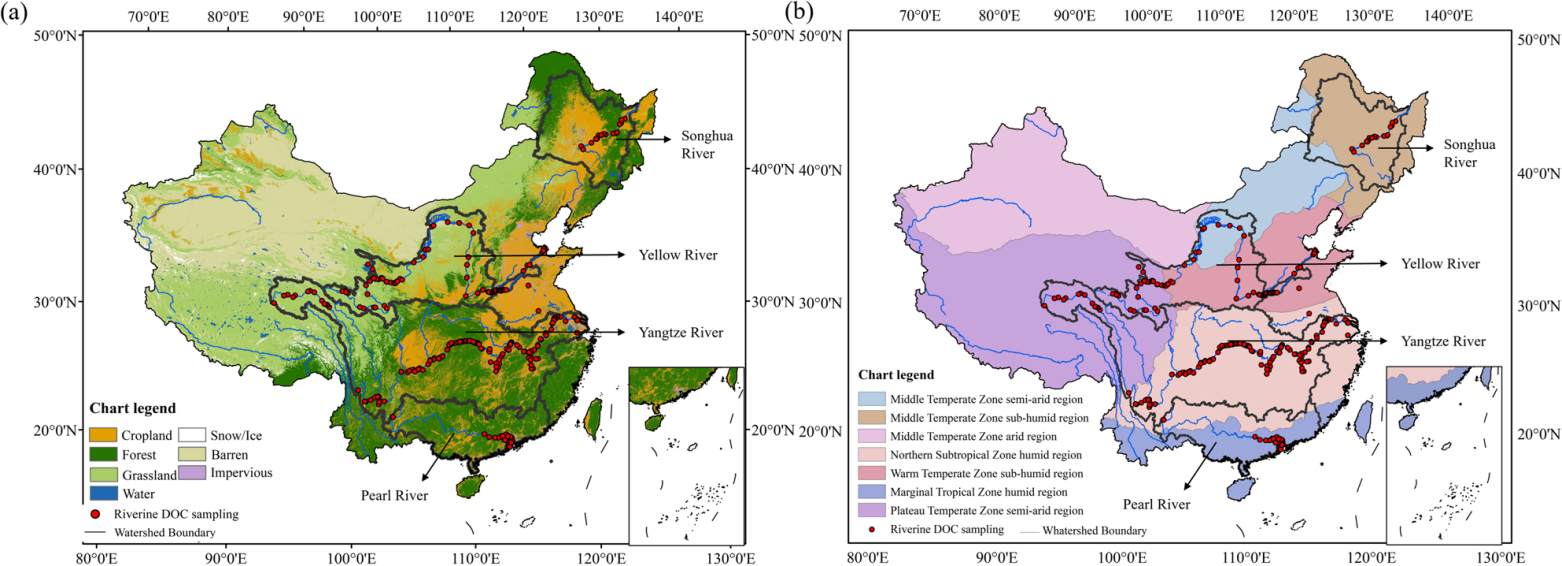
Spatial distribution of the study areas and in situ DOC sampling sites in four major river basins of China (i.e., the Songhua River Basin, Yellow River Basin, Yangtze River Basin, and Pearl River Basin). (**a**) Distribution of land use types, with watershed boundaries and riverine DOC sampling sites overlaid; (**b**) Distribution of China’s climate zones, with watershed boundaries and riverine DOC sampling sites overlaid

This study focused on four major river basins in China: the Songhua River Basin, Yellow River Basin, Yangtze River Basin, and Pearl River Basin (Fig. 1), which span diverse climatic zones and cover China’s primary land use types. Specifically, the Songhua River Basin in northeastern China is located in the mid-temperate semi-humid region, features cropland as its predominant land use (45%), with substantial forest coverage (42%), alongside extensive black soil regions. Moreover, this basin experiences intensive farming and industrial activities. The Yellow River Basin is mainly located in the warm-temperate semi-humid region and mid-temperate semi-arid region, and flows predominantly through regions of cropland and grassland with severe soil erosion. The Yangtze River Basin, as China’s largest basin, is mainly situated in the north-subtropical humid region with abundant precipitation. This basin has diverse land use types that include cropland, forests, water areas, and urban areas. With a dense population, numerous cities, and a rich diversity of human activities, the basin is one of China’s most economically and culturally developed regions. The Pearl River Basin in southern China is located in the marginal tropical humid region with abundant precipitation and dense river networks. Its land use is dominated by agriculture and forest with intense human activities. The diversity and representativeness of these basins make them ideal for investigating the spatiotemporal variations in DOC in China’s major rivers, and for offering insights into their connections with climate and human activities. For detailed quantitative characteristics of each of the four basins, please refer to Table S1 and Table S2.

### Data acquisition

For this study, C_DOC_ data were obtained through a systematic literature review of Chinese and English publications on the CNKI and Web of Science. Criteria for inclusion were as follows: field-sampled data with clear coordinates and sampling times; use of filter membranes with a pore size of 0.22–0.7 μm; [3] DOC content convertible to concentration (unit: mg L⁻¹, carbon basis); and sampling of surface or mixed water to exclude stratification effects. Data were extracted from the main text, figures, tables, and supplementary materials of eligible studies. For papers without original data, the GetData Graph Digitizer (ver2.25) software was used to digitalize graphs to extract the necessary data. Ultimately, 127 valid papers were consolidated, from which 1922 groups of C_DOC_ data from 1997 to 2023 were extracted. The specific quantitative characteristics of C_DOC_ in each river basin are summarized in Table 1.

**Table 1:** Quantitative characteristics of C_DOC_ data in four major river basins

River Basin	River Reach	Number of Observations	Temporal Coverage	Coverage of Major Hydrological Periods
SHR	Middle	25	2008–2018	Wet, Normal, Dry
SHR	Lower	56	2008–2014	Wet, Normal, Dry
YR	Upper	41	2003–2019	Wet, Normal
YR	Middle	164	2003–2019	Wet, Normal, Dry
YR	Lower	186	2003–2019	Wet, Normal, Dry
YZR	Upper	53	2007–2021	Wet, Normal, Dry
YZR	Middle	193	1997–2021	Wet, Normal, Dry
YZR	Lower	298	1998–2021	Wet, Normal, Dry
PR	Middle	22	2008–2018	Wet, Normal, Dry
PR	Lower	395	2008–2014	Wet, Normal, Dry

In this study, land use types were derived from the 30-m Chinese Land Cover Dataset (1985–2023) published by the Yang Jie and Huang Xin team at Wuhan University (https://zenodo.org/records/12779975) [30]. The F_DOC_ data were calculated by multiplying streamflow data by C_DOC_. The streamflow data were obtained from the River discharge and related historical data from the Global Flood Awareness System (v 4.0) (10.24381/cds.a4fdd6b9) [31]. Data for two climatic factors were obtained from separate datasets: 1-km monthly precipitation dataset for China (1901–2024) (10.5281/zenodo.3114194) [32–36] and 1-km monthly mean air temperature dataset for China (1901–2024) (10.11888/Meteoro.tpdc.270961) [33–37]. In addition to land use type changes, this study also selected two human activity factors: population density and GDP. Population density data were sourced from the Chinese Population Spatial Distribution Kilometer Grid Dataset (https://www.resdc.cn/DOI/DOI.aspx?DOIID=32) [38], and GDP data were extracted from the Chinese GDP Spatial Distribution Kilometer Grid Dataset (https://www.resdc.cn/DOI/DOI.aspx?DOIID=33) [39]. The hydroelectric power station and reservoir data were derived from a global dataset combining open-source hydropower plant and reservoir data (10.1038/s41597-025-04975-0) [40]. The soil organic carbon data were obtained from a China dataset of soil properties for land surface modelling (version 2, CSDLv2) (10.5194/essd-17-517-2025) [41]. The detailed information of the above data is provided in Table 2.

**Table 2:** Data sources used in this study

Data name	Time span	Temporal Resolution	Spatial Resolution	Data sources
Streamflow	1979–2024	Daily	5 km	European Commission, Joint Research Centre
Precipitation	1901–2023	Monthly	1 km	National Tibetan Plateau/Third Pole Environment Data Center
Air Temperature	1901–2023	Monthly	1 km	National Tibetan Plateau/Third Pole Environment Data Center
Land Use	1985–2023	Yearly	30 m	[30]
Population	1995–2020	Five-year	1 km	Center for Resources and Environmental Sciences and Data
GDP	1995–2020	Five-year	1 km	Center for Resources and Environmental Sciences and Data
Hydroelectric Power Station and Reservoir	1979–2021	/	/	National Earth System Science Data Center
Soil Organic Carbon	/	/	90 m	[41]

### Data pretreatment

To define the spatial boundaries for environmental variable extraction, the upstream catchment area for each sampling site was delineated based on the vector river network topology provided by the GRADES dataset [42, 43]. This dataset is constructed using the RAPID vector river routing model derived from the 90-m MERIT Hydro hydrologically corrected topography data. Specifically, the topological relationships within the dataset were utilized to recursively trace all river reaches upstream of the sampling locations. The unit catchments corresponding to these identified reaches were then spatially merged to generate complete upstream catchment polygons.

The meteorological (precipitation and air temperature), land use type, GDP, population density, and soil organic carbon content data for each collected sample were obtained from the mean value and standard error within the upstream catchment area of the sampling site. Additionally, the number of hydroelectric power stations and reservoirs within the upstream catchment was quantified. If the temporal resolution of the data for certain influencing factors did not match the DOC sampling timeline, the data closest to the sampling time was selected for temporal alignment. The F_DOC_ and DOC yield was calculated using the following equation:

1 $$\:{F}_{DOC}={C}_{DOC}\times\:Streamflow$$

2 $$\:DOC\:yield={F}_{DOC}\div \:Basin\:Area$$

### Methods

#### Mann–Kendall trend analysis

This study employed Mann–Kendall trend analysis and the Sen’s slope estimator to assess the interannual trends of C_DOC_ and F_DOC_.

The nonparametric Mann–Kendall trend analysis test is used to verify the statistical significance of a monotonic trend in a time series through the following procedure. First, it postulates a null hypothesis (H_0_) of no trend in the time series and an alternative hypothesis (H_1_) indicating the existence of a monotonic trend. Then, it calculates the sign function $\:sgn({x}_{j}-{x}_{i})$ to define the statistic $\:S$ as follows:

3 $$\:S=\sum_{k=1}^{n-1}\sum_{j=i+1}^{n}sgn({x}_{j}-{x}_{i})$$

where $\:{x}_{j}$ and $\:{x}_{i}$ represent the values in year $\:i$ and year $\:j$, respectively. The $\:sgn$ function returns 1, 0, or − 1 depending on whether $\:{x}_{j}$ is greater than, equal to, or less than $\:{x}_{i}$, respectively. Finally, when duplicate values exist, the variance $\:Var\left(S\right)$ is corrected, and the standardized statistic $\:Z$ is calculated. If $\:\left|Z\right|\ge\:1.96$ (corresponding to $\:\alpha\:=0.05$), then H_0_ is rejected, indicating a significant trend ($\:p<0.05$).

4 $$\:Z=\left\{\begin{array}{cc}\frac{S-1}{\sqrt{Var\left(S\right)}}&\:S>0\\\:0&\:S=0\\\:\frac{S+1}{\sqrt{Var\left(S\right)}}&\:S<0\end{array}\right.,$$

5 $$\:{Sen}_{slope}=Median\left(\frac{{x}_{j}-{x}_{i}}{j-i}\right)\left(i<j\right)$$

Integration of Sen’s slope for trend rate estimation and the Mann–Kendall test for statistical significance assessment is well-suited for long-term trend analysis of non-normally distributed data. In this study, trends in C_DOC_ and F_DOC_ were considered statistically significant when $\:p<0.05$.

#### Uncertainty characterization and data standardization

In this study, the uncertainty range was characterized via the 95% confidence interval ($\:CI$) to reflect fluctuations in C_DOC_, F_DOC_, and streamflow. The formula is presented as follows:

6 $$\:CI=\stackrel{-}{x}\pm\:1.96\times\:\frac{\sigma\:}{\surd\:n},$$

where $\:\stackrel{-}{x}$ denotes the mean value, $\:\sigma\:$ denotes the standard deviation, and $\:n$ denotes the sample size.

 Seasonal normalization was performed by normalizing monthly values ($\:{x}_{norm,t}$) of C_DOC_, F_DOC_, and streamflow to 0–1 employing the following formula:

7 $$\:{x}_{norm,t}=\frac{{x}_{t}-{x}_{min}}{{x}_{max}-{x}_{min}}$$

where $\:{x}_{t}$ represents the value in month $\:t$, and $\:{x}_{max}$ and $\:{x}_{min}$ denote the maximum and minimum values of the entire time series, respectively.

This approach enables comparison of the seasonal phase and amplitude variations across different variables.

#### Significance test of difference

To quantify whether the differences in C_DOC_ and F_DOC_ between different spatial units (different river basins, different reaches within the same river basin) are statistically significant, this study adopted the Kruskal-Wallis test for significance analysis.

During the test, “river basins” (Songhua River Basin, Yellow River Basin, Yangtze River Basin, Pearl River Basin) and “river reaches” (upper reach, middle reach, lower reach of each basin) were used as grouping variables, respectively, and independent tests were conducted on the two core indicators of C_DOC_ and F_DOC_. The significance level was set at *p* < 0.05: if the test result was *p* < 0.05, it indicated that the difference in C_DOC_ or F_DOC_ under this grouping dimension (e.g., between different basins, between different reaches of the same basin) was statistically significant; if *p* ≥ 0.05, it indicated that the difference was not statistically significant.

#### Structural equation modeling

In this study, SEM was employed to analyze the statistical relationships among variables [44]. SEM is a powerful analytical tool that integrates multiple regression with factor analysis. This integration allows estimation of both direct and indirect effects among variables. Consequently, it is well suited for evaluating complex models that include multiple interdependent variables, as well as latent variables inferred from other observations.

In our SEM analysis, we employed the R 4.0.2 computing environment and the “plspm” package [45]. To identify the influencing factors of the spatiotemporal variations in DOC for integration into a combined model and systematically evaluate their paths of influence on DOC, this study systematically explored the correlations between C_DOC_, F_DOC_, and various potential influencing factors through a Spearman correlation analysis. During the analysis, a significance level of *p* < 0.05 was used as the criterion, and variables that showed a significant correlation with C_DOC_ or F_DOC_ were screened out and identified as the core influencing factors driving the spatiotemporal variations in DOC. The specific categories and included indicators of the aforementioned potential influencing factors are presented in Table 3.

**Table 3:** Potential influencing factors and their included indicators

Potential Influencing Factors	Included Indicators
Climatic factors	Precipitation, Air temperature
Human activity factors	Gross Domestic Product (GDP), Population density, Hydroelectric power stations and reservoirs
Land use types	Cropland (%), Forest (%), Shrub (%), Grassland (%), Water (%), Snow_Ice (%), Barren (%), Impervious (%), Wetland (%)
Soil factor	Soil organic carbon

## Results

### Spatial and temporal distributions of C_DOC_ in China’s major rivers

**Fig. 2:**
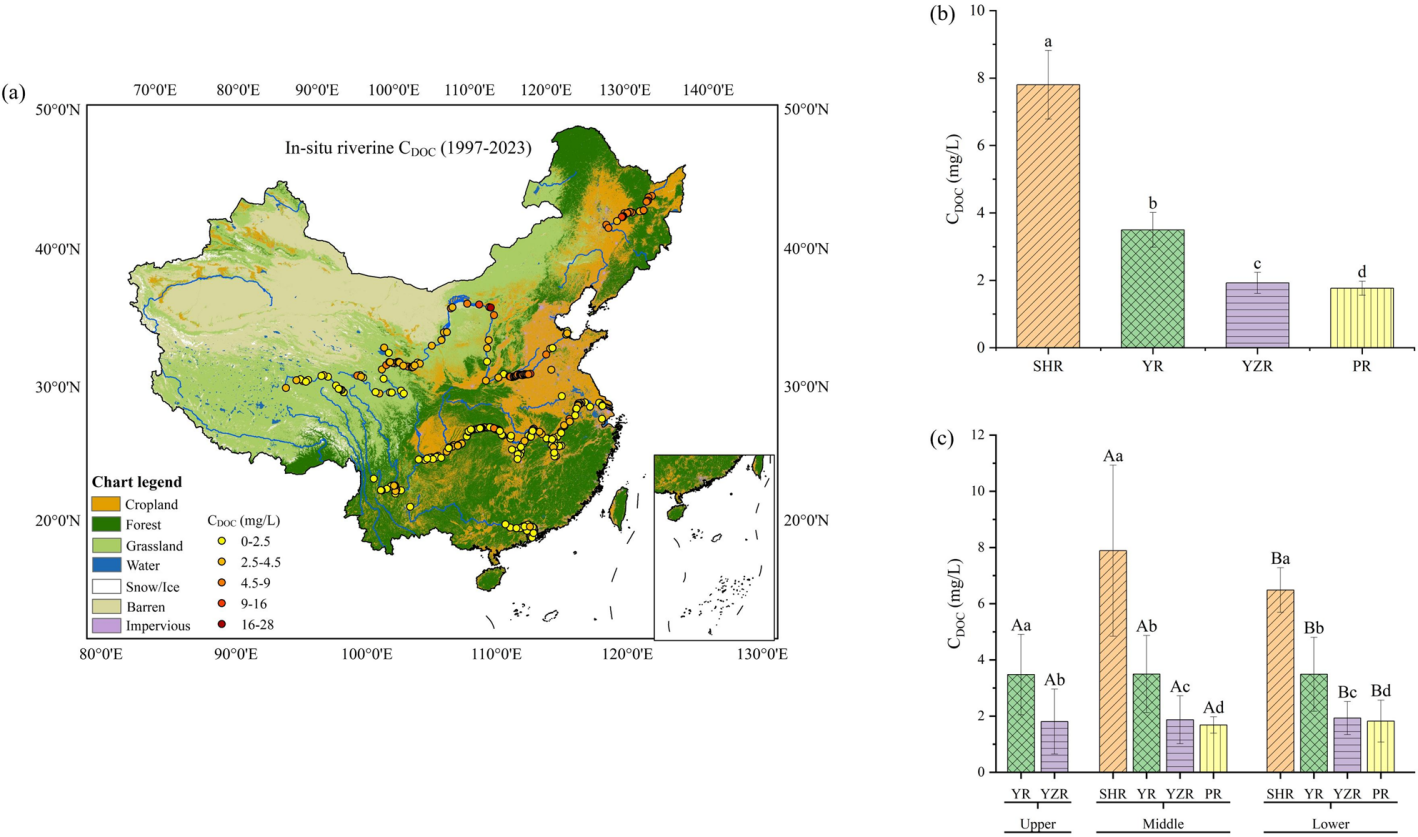
Spatial distribution characteristics of C_DOC_ in China’s four major river basins (**a**) Multiyear (1997–2023) spatial distribution of C_DOC_; (**b**) Multiyear (1997–2023) mean values of C_DOC_ in each river basin; (**c**) Distribution variations of C_DOC_ in the upper, middle, and lower reaches. In (**b**) and (**c**), both lowercase and uppercase letters are based on the Kruskal-Wallis test: lowercase letters indicate the significance of differences among different river basins, while uppercase letters indicate the significance of differences between the upper, middle, and lower reaches within the same river basin; the same letter means no significant difference, and different letters mean significant difference. (SHR: Songhua River Basin, YR: Yellow River Basin, YZR: Yangtze River Basin, and PR: Pearl River Basin)

Figure 2 illustrates the spatial distribution of C_DOC_ across the four representative river basins in China. Marked spatial heterogeneity was observed in C_DOC_ among the four rivers, with distinct differences across basins. The Songhua River Basin exhibited the highest C_DOC_, with an average value of 7.85 ± 1.01 mg L⁻¹. The Yellow River Basin recorded an average C_DOC_ value of 3.50 ± 0.51 mg L⁻¹. The Yangtze River Basin and Pearl River Basin averaged 1.92 ± 0.31 and 1.77 ± 0.21 mg L⁻¹, respectively, both below the global riverine average of 5.75 mg L⁻¹ [46]. Overall, C_DOC_ in the four major Chinese rivers demonstrated a north–south gradient with higher concentrations observed in the northern regions. Figure 2c further shows the spatial heterogeneity of C_DOC_ across the upper, middle, and lower reaches of these basins. In the Songhua River Basin, C_DOC_ peaked at 7.89 ± 3.04 mg L⁻¹ in the midstream and diminished to 6.49 ± 0.79 mg L⁻¹ downstream. In the Yellow River Basin, C_DOC_ exhibited minor fluctuations while remaining relatively stable: 3.48 ± 1.43 mg L⁻¹ in the upper reaches, a slight increase to 3.50 ± 1.37 mg L⁻¹ in the middle reaches, and a subsequent drop to 3.49 ± 1.32 mg L⁻¹ in the lower reaches. The average C_DOC_ values in the upstream, midstream, and downstream reaches of the Yangtze River Basin were 1.81 ± 1.15, 1.87 ± 0.85, and 1.93 ± 0.58 mg L⁻¹, respectively. Among them, there was no significant difference in C_DOC_ between the upstream and midstream reaches (*p* > 0.05), whereas the C_DOC_ in the downstream reach significantly increased compared with that in the upstream and midstream reaches (both *p* < 0.05). In the Pearl River Basin, C_DOC_ exhibited a progressive increase along the river channel, rising by 8% from 1.68 ± 0.29 mg L⁻¹ in the midstream to 1.82 ± 0.74 mg L⁻¹ downstream. Overall, the longitudinal patterns of C_DOC_ displayed diverse characteristics across the different basins, highlighting the need for further research into its influencing factors and associated mechanisms.

**Fig. 3:**
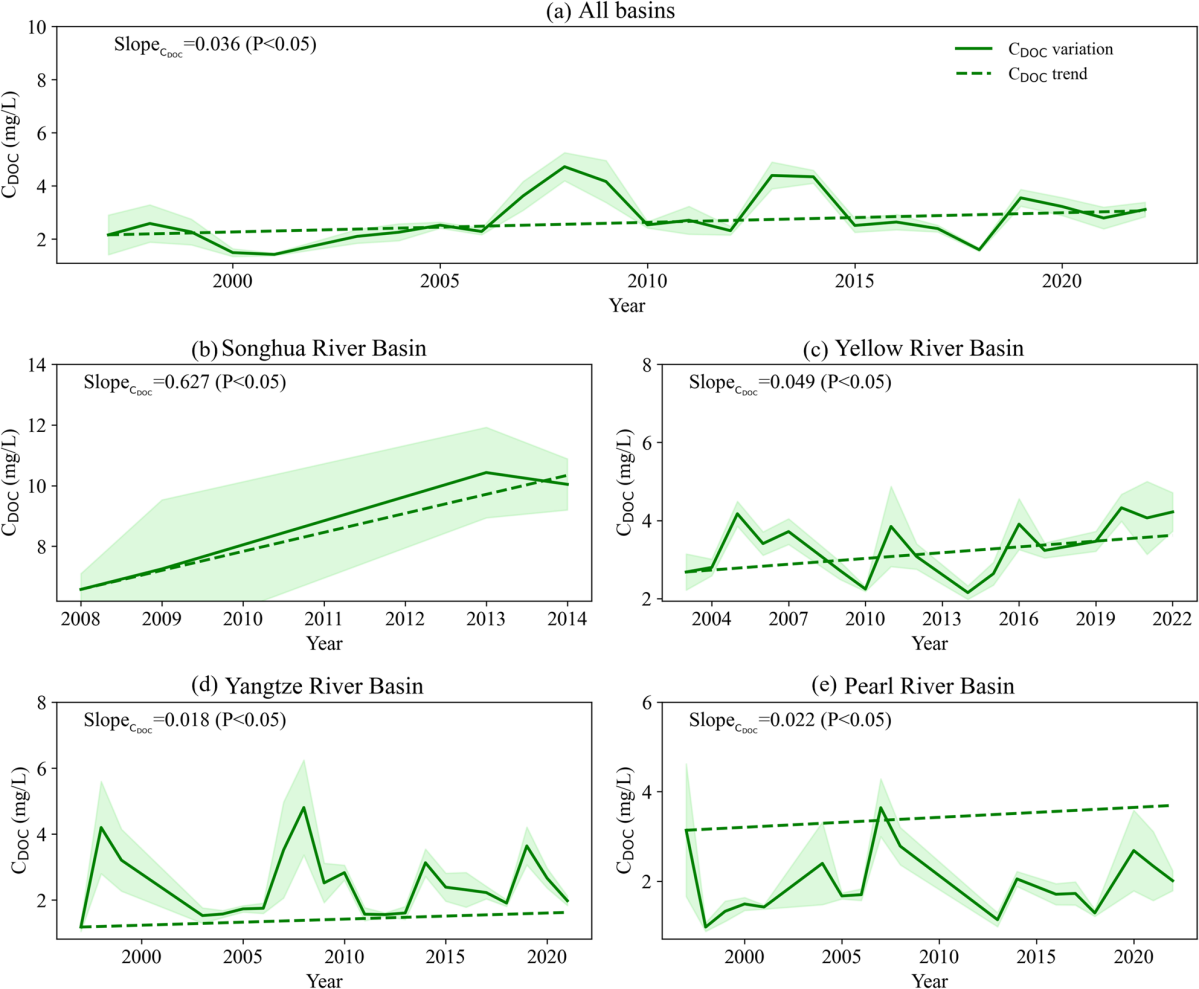
Temporal variations and trends in C_DOC_ in (**a**) all basins, (**b**) Songhua River Basin, (**c**) Yellow River Basin, (**d**) Yangtze River Basin, and (**e**) Pearl River Basin. Trend magnitudes were calculated using the Sen’s slope estimator, and their statistical significance was assessed using the Mann–Kendall test. Color shading represents uncertainties with 95% confidence intervals

**Fig. 4:**
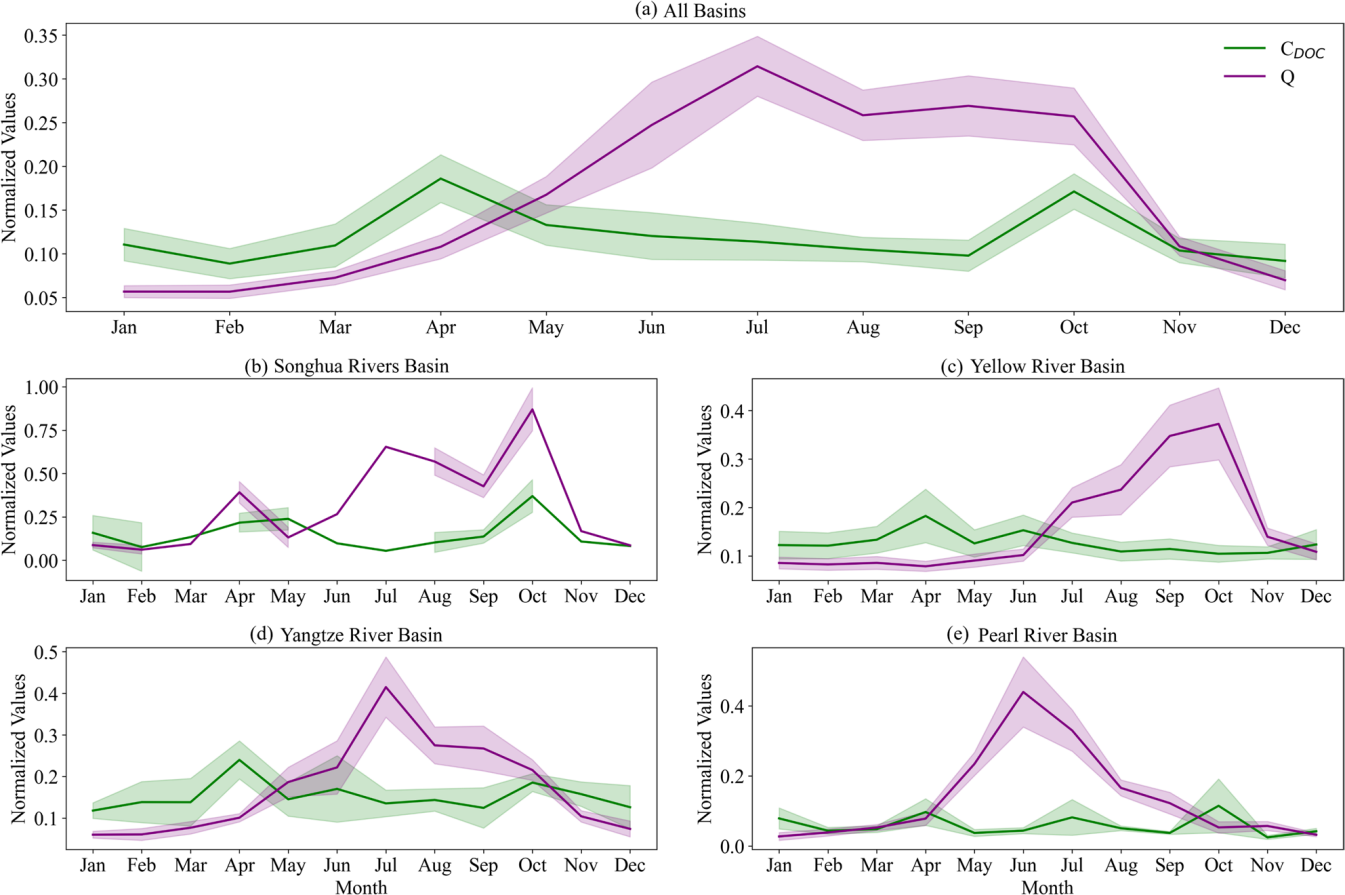
Normalized monthly values of C_DOC_ and streamflow in (**a**) all basins, (**b**) Songhua River Basin, (**c**) Yellow River Basin, (**d**) Yangtze River Basin, and (**e**) Pearl River Basin. Range normalization to values of 0–1 was employed, based on the min–max values of each variable. Color shading represents uncertainties with 95% confidence intervals

Beyond spatial patterns, the temporal dynamics of C_DOC_ in the four major river basins also exhibited notable characteristics, as presented in Figs. 3 and 4. During 1997–2023, remarkable temporal trends of C_DOC_ were observed. At the national scale, C_DOC_ increased significantly at an annual rate of 0.04 mg L⁻¹ (*p* < 0.05). From the perspective of individual basins, C_DOC_ showed a statistically significant upward trend in all four rivers (*p* < 0.05). In terms of seasonal variations, C_DOC_ exhibited distinct patterns during the study period, with marked differences across the basins. Typically, C_DOC_ peaked in spring, dropped to its annual minimum in summer, and gradually increased to a minor peak in autumn. The spring peak in C_DOC_ suggests that it might be influenced more by early spring environmental conditions such as vegetation growth and soil thawing.

### Spatial and temporal distributions of F_DOC_ in china’s major rivers

**Fig. 5:**
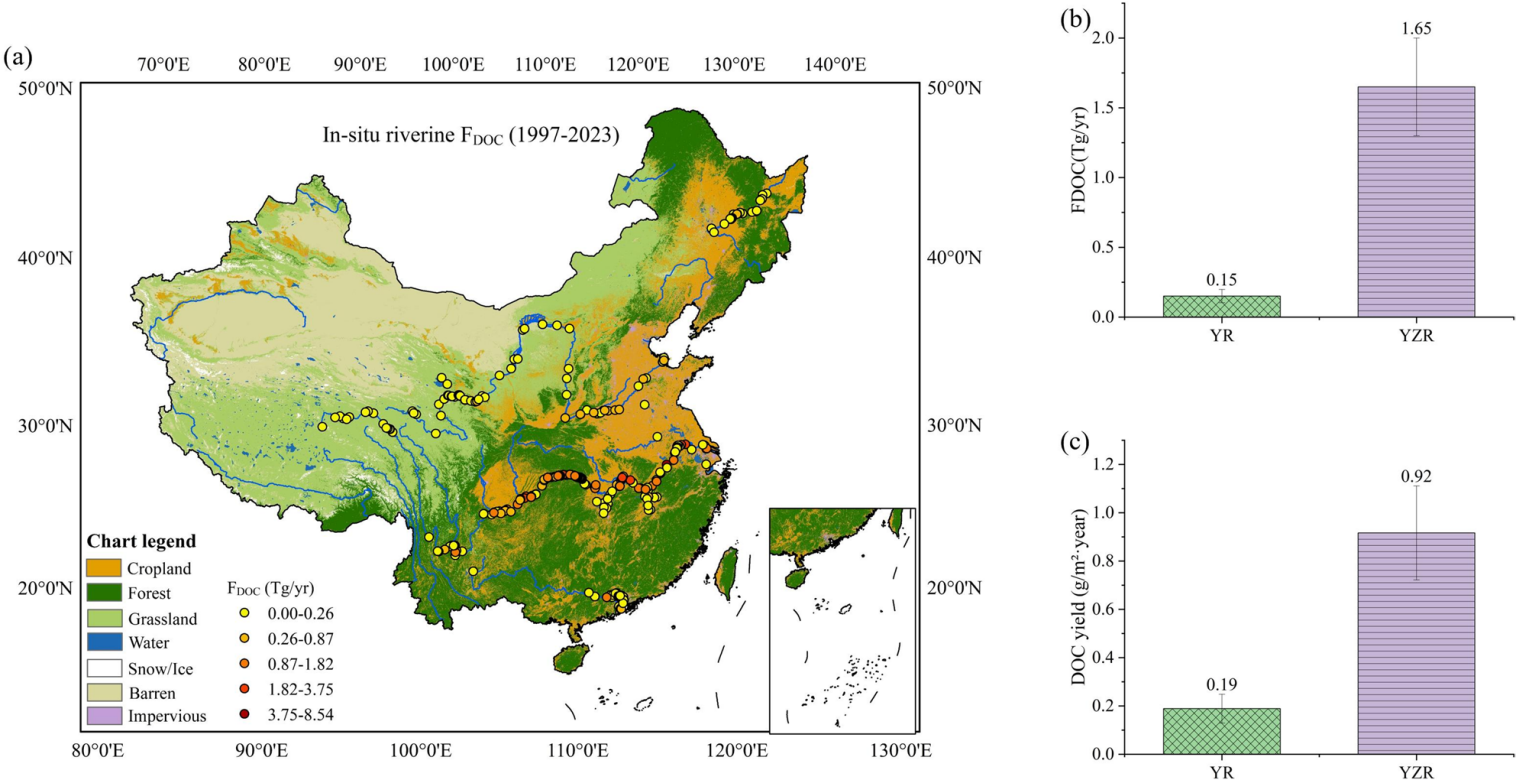
Spatial distribution characteristics of F_DOC_ in China’s major river basins (**a**) Multiyear (1997–2023) spatial distribution of F_DOC_; (**b**) Multiyear (1997–2023) mean values of F_DOC_ at the estuaries of the Yangtze River and the Yellow River; (**c**) Multiyear (1997–2023) mean values of ADF in Yangtze River Basin and Yellow River Basin

Figure 5 illustrates the spatial distribution characteristics of F_DOC_ in China’s major river basins. As shown in Fig. 5a, F_DOC_ exhibited a south-high and north-low spatial pattern. Additionally, the F_DOC_ flux at the Yangtze River Estuary (1.65 ± 0.35 Tg yr⁻¹) was significantly higher than that at the Yellow River Estuary (0.15 ± 0.04 Tg yr⁻¹) (Fig. 5b). This study further calculated the DOC flux per unit area (i.e., DOC yield) of the Yangtze River and the Yellow River, which also displayed a consistent spatial differentiation pattern: the DOC flux per unit area of the Yangtze River (0.92 ± 0.19 g m⁻² yr⁻¹) was notably higher than that of the Yellow River (0.19 ± 0.06 g m⁻² yr⁻¹) (Fig. 5c). However, the magnitude of the difference between the two rivers in this indicator was smaller than that in the F_DOC_ flux at the estuaries.

**Fig. 6:**
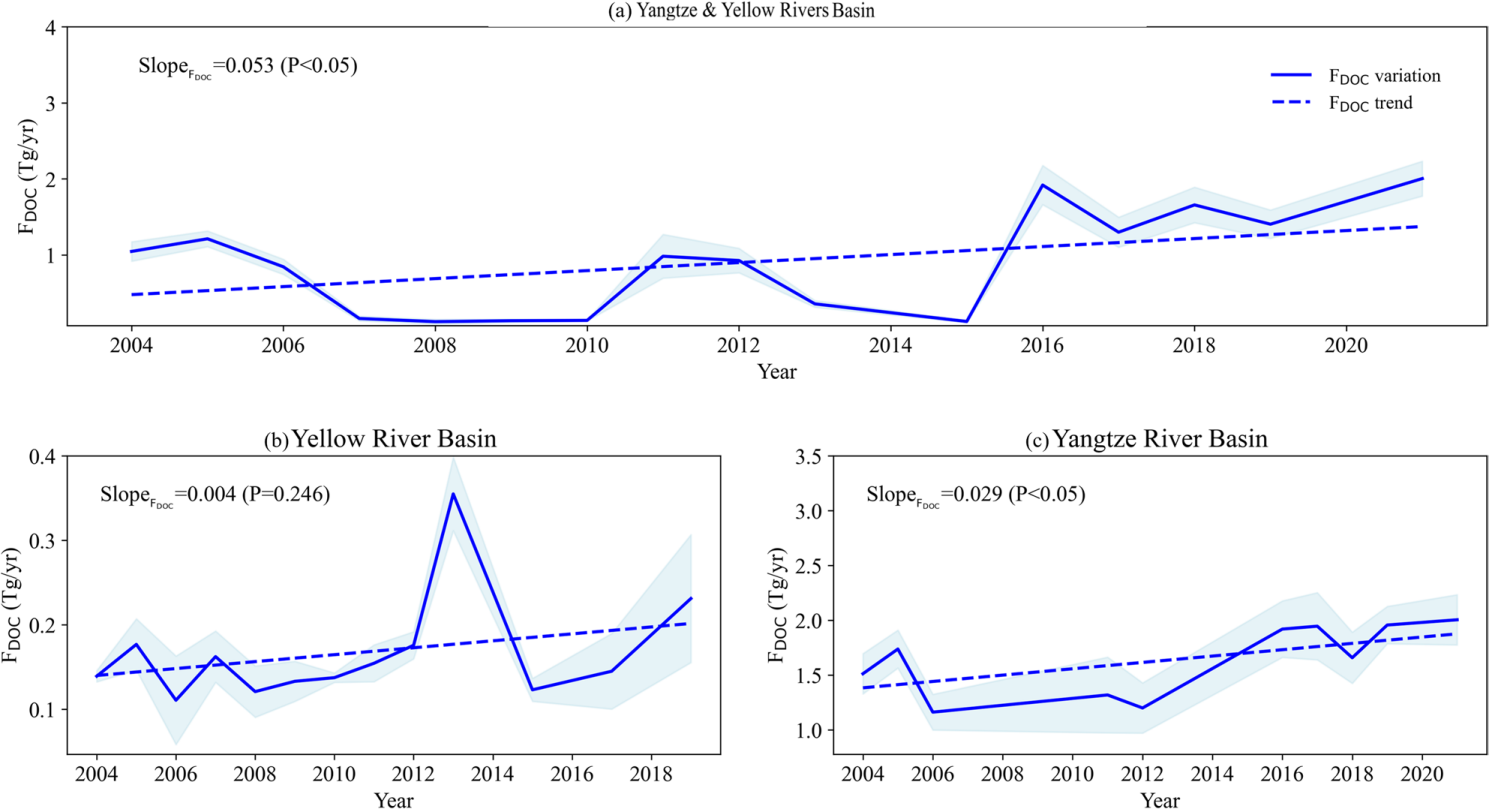
Temporal variations and trends in F_DOC_ in the estuaries of the (**a**) Yangtze and Yellow River Basins, (**b**) Yellow River Basin, (**c**) Yangtze River Basin. Trend magnitudes were calculated using the Sen’s slope estimator, and their statistical significance was assessed using the Mann–Kendall test. Color shading represents uncertainties with 95% confidence intervals

**Fig. 7:**
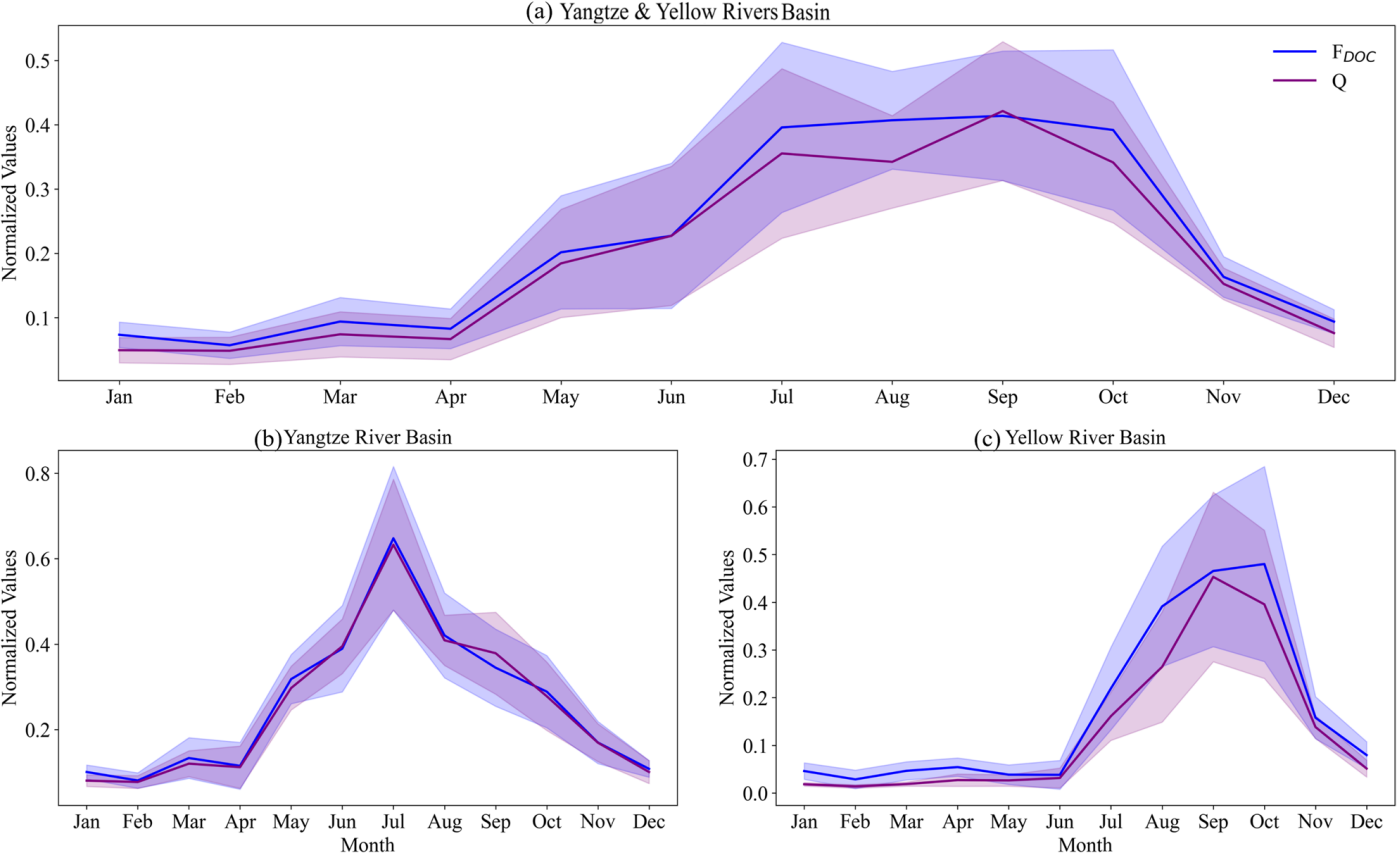
Normalized monthly values of F_DOC_ in the estuaries, and streamflow of the (**a**) Yangtze and Yellow River Basins, (**b**) Yellow River Basin, (**c**) Yangtze River Basin. Range normalization to values of 0–1 was employed, based on the min–max values of each variable. Color shading represents uncertainties with 95% confidence intervals

During the period 1997–2023, the F_DOC_ into the sea from China’s two largest rivers, the Yangtze River and the Yellow River, exhibited a significant upward trend. Its annual growth rate was 0.05 Tg yr⁻¹ (*p* < 0.05) (Fig. 6a). From the perspective of individual river basins, the F_DOC_ into the sea from the Yangtze River Basin showed a statistically significant upward trend (*p* < 0.05), whereas the upward trend of the F_DOC_ into the sea from the Yellow River Basin was not statistically significant (Fig. 6b, c). Additionally, as shown in Fig. 7, F_DOC_ exhibited distinct seasonal variations during the study period: its variations aligned closely with streamflow trends, featuring a distinct seasonal pattern characterized by a peak during the rainy season and subsequent decline during the dry season; specifically, during the summer months with higher precipitation and greater streamflow, F_DOC_ levels increased, while during the dry winter season, F_DOC_ levels diminished. This seasonal pattern implies that F_DOC_ is closely associated with precipitation and streamflow dynamics.

### Spearman correlation analysis of C_DOC_ and F_DOC_ with impact factors

**Fig. 8:**
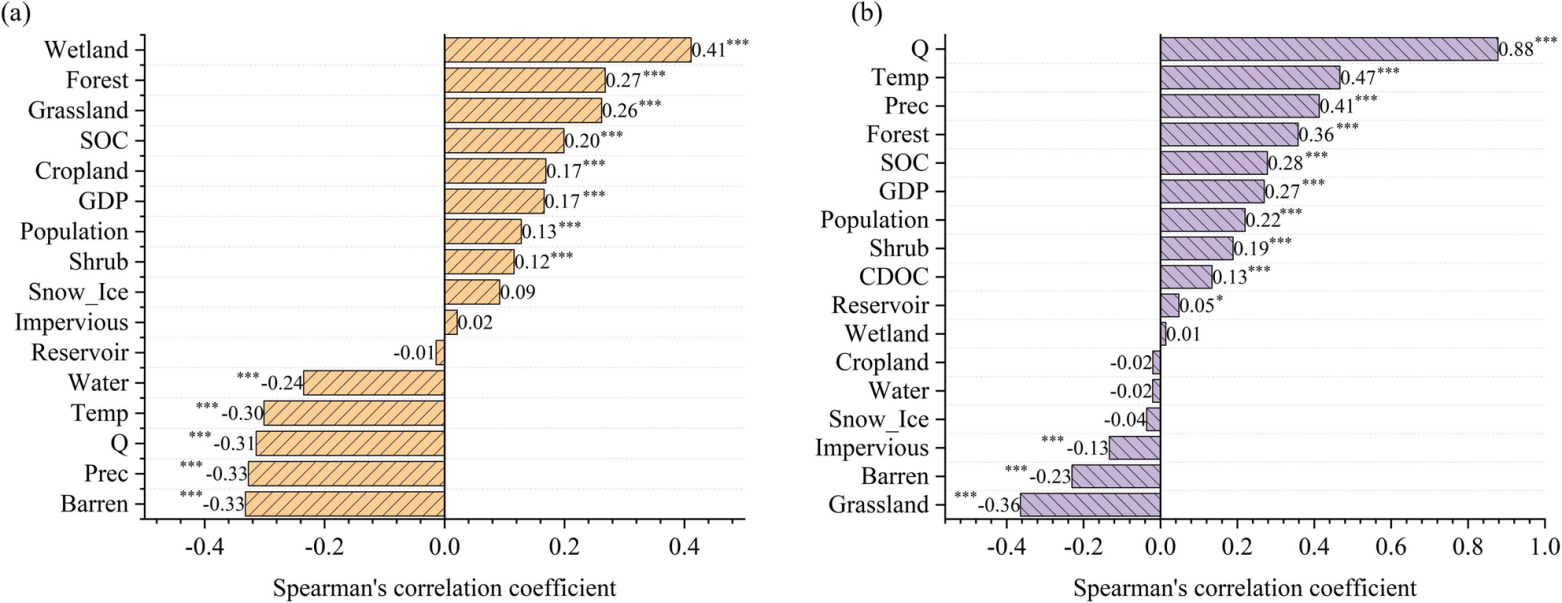
Results of Spearman correlation analysis. (**a**) Results of Spearman correlation analysis between C_DOC_ and other variables; (**b**) Results of Spearman correlation analysis between F_DOC_ and other variables. Note on significance: *** are significant at *p* < 0.001, ** at *p* < 0.01, and * at *p* < 0.05

The Spearman correlation coefficient (rₛ) analysis results of C_DOC_ with various environmental variables, land use types, and anthropogenic activity variables showed obvious differences in correlations (Fig. 8a). Among them, C_DOC_ had a significantly moderately positive correlation with wetland (rₛ=0.41, *p* < 0.05), and significantly weakly positive correlations with forest (rₛ=0.27, *p* < 0.05) and grassland (rₛ=0.26, *p* < 0.05). It also exhibited significant weak positive correlations with SOC, cropland, GDP, population, shrub, and snow_ice (rₛ=0.20, 0.17, 0.17, 0.13, 0.12, 0.09, respectively, *p* < 0.05). In contrast, C_DOC_ had significantly moderately negative correlations with barren, precipitation (Prec), streamflow (Q), and temperature (Temp) (rₛ=−0.33, −0.32, −0.31, −0.30 respectively, *p* < 0.05), and a significantly weakly negative correlation with water (rₛ=−0.24, *p* < 0.05). In addition, the correlations between C_DOC_ and reservoir, as well as impervious, were not statistically significant (*p* > 0.05). Among all variables, C_DOC_ had the strongest positive correlation with wetland and the strongest negative correlation with barren.

The Spearman correlation analysis results of F_DOC_ with various variables indicated significant differences from C_DOC_ (Fig. 8b). F_DOC_ was significantly strongly positively correlated with Q (rₛ=0.88, *p* < 0.05) and moderately positively correlated with temperature and precipitation (rₛ=0.47, 0.41 respectively, *p* < 0.05). It was also significantly weakly positively correlated with forest, SOC, GDP, population, shrub, and C_DOC_ (rₛ=0.36, 0.26, 0.27, 0.22, 0.19, 0.13 respectively, *p* < 0.05). On the contrary, F_DOC_ was significantly moderately negatively correlated with grassland (rₛ=−0.36, *p* < 0.05) and weakly negatively correlated with barren and impervious (rₛ=−0.23, −0.13 respectively, *p* < 0.05). In addition, the correlations between F_DOC_ and snow_ice, water, cropland and wetland were not statistically significant (*p* > 0.05). Among all variables, F_DOC_ had an extremely strong positive correlation with runoff and the strongest negative correlation with grassland.

### Impact of climate change and human activities on DOC dynamics

**Fig. 9:**
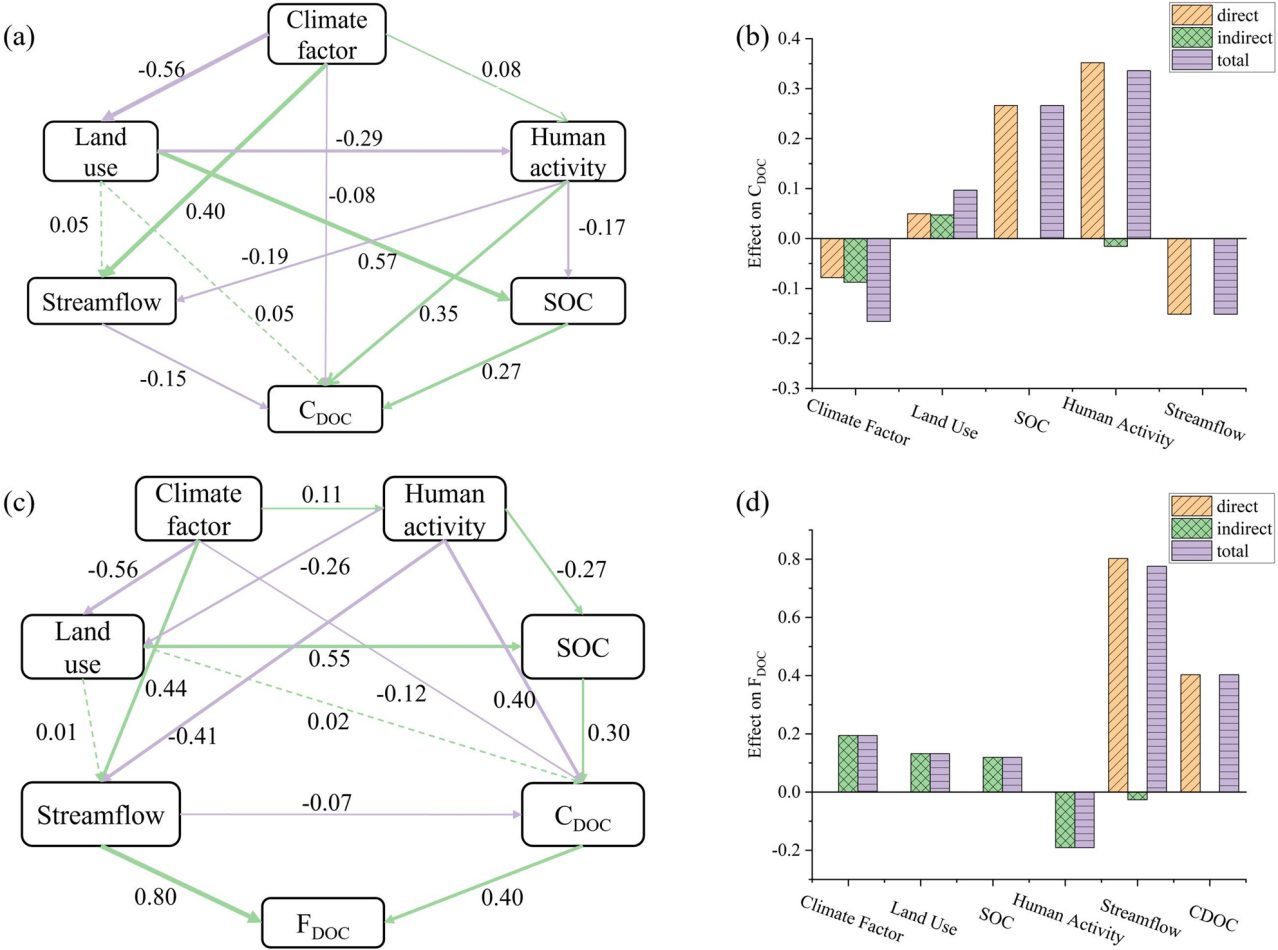
Path effects of SEM on DOC dynamics in the studied major rivers of China: (**a**) Path coefficient strength and its significance for C_DOC_ (purple/green solid arrows indicate significant positive/negative effects at *p* < 0.05, dashed arrows show nonsignificant relationships, and arrow width is proportional to standardized path coefficients with labeled values); (**b**) Path coefficient strength and its significance for F_DOC_ (annotation rules same as a); (**c**) Path effects of climate factors, land use types, streamflow, and human activity factors on C_DOC_; (**d**) Path effects of climate factors, land use types, streamflow, human activity factors, and C_DOC_ on F_DOC_

This study employed SEM to analyze the complex interactions among climate, streamflow, human activities, land use types, SOC, and DOC, and the results are presented in Fig. 9. The results suggest that climate change influenced DOC dynamics through both direct and indirect pathways. For C_DOC_, climate factors exhibited a significant direct negative effect (direct effect: −0.08), coupled with a stronger indirect negative effect (−0.09) by inhibiting land use (−0.56) and promoting streamflow (0.40), resulting in a total effect of −0.17. In contrast, for F_DOC_, climate factors exerted a significant positive indirect effect (0.19) primarily by promoting streamflow (0.44), with a total effect of 0.19.

Human activities had a moderate positive impact on C_DOC_ but an indirect negative effect on F_DOC_. For C_DOC_, human activities showed a significant direct positive effect (0.35), which is likely attributable to wastewater discharge and agricultural pollution that directly increase C_DOC_ input, and a weak indirect negative effect (−0.02) by regulating streamflow and soil organic carbon, leading to a total effect of 0.33. For F_DOC_, human activities collectively produced an indirect negative effect (−0.19) through multiple indirect pathways, with a total effect of −0.19, the magnitude of which is nevertheless smaller in absolute terms than the total effect on C_DOC_.

Other factors, including land use, soil organic carbon, streamflow, and C_DOC_ itself, also played non-negligible roles. For C_DOC_, among these factors, SOC emerged as the most prominent positive contributor (total effect: 0.26), driven entirely by its direct effect. Land use exerted a non-significant positive total effect (0.10, *p* > 0.05). In contrast, streamflow acted as a major inhibitory factor (total effect: −0.15) due to its significant direct negative effect (−0.15). For F_DOC_, streamflow was the core driving factor with the strongest positive total effect (0.78), where its highly significant direct positive effect (0.80) served as the primary contribution source. C_DOC_ emerged as the secondary positive factor (total effect: 0.40) via its significant direct positive effect (0.40, *p* < 0.05); land use and SOC exerted weak positive total effects (0.13 and 0.12, respectively) through indirect pathways.

## Discussion

### Driving mechanisms behind the Spatial differentiation of DOC

This study integrated the results of SEM to analyze the impacts of climate change and human activities on the spatial distributions of C_DOC_ and F_DOC_ in four major rivers in China. The differing spatial distribution patterns of C_DOC_ and F_DOC_ reflected the synergistic interactions between natural geographical conditions and anthropogenic activities. In the studied Chinese rivers, C_DOC_ declined with latitude (Fig. 2), aligning with the findings of Dai et al. [18]. This trend might be attributable to the dilution effect of abundant precipitation in humid subtropical regions of the Northern Hemisphere. Our results also indicate that C_DOC_ is significantly negatively correlated with precipitation (Fig. 8a). Additionally, this pattern might also be closely linked to the widespread presence of permafrost in high-latitude northern regions, higher soil organic matter accumulation, and enhanced carbon release through permafrost thawing caused by global warming [47–50]. Both the SEM results (Fig. 9) and the Spearman correlation analysis results (Fig. 8a) indicate that climate and SOC are primary drivers of C_DOC_ variations. Notably, despite lower C_DOC_ levels in the southern rivers, their substantially higher streamflow led to obviously higher values of F_DOC_ (Fig. 5). This highlights the dominant role of streamflow in determining F_DOC_, with a total effect of 0.77 on F_DOC_, as shown in Fig. 9d.

**Fig. 10:**
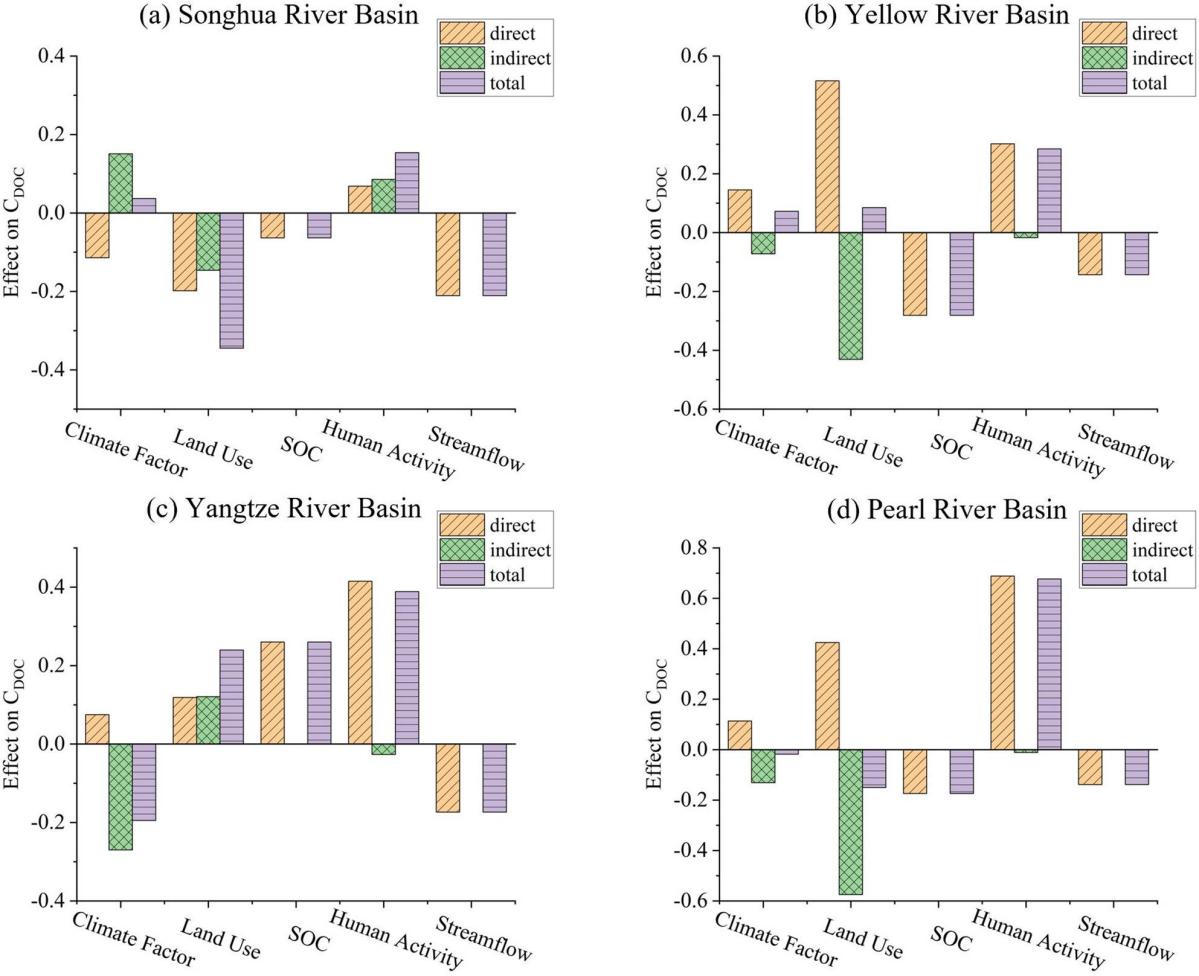
Path effects of climate factors, land use types, SOC, human activity, and streamflow factors on C_DOC_: (**a**) the Songhua River Basin, (**b**) the Yellow River Basin, (**c**) the Yangtze River Basin, and (**d**) the Pearl River Basin

Furthermore, this study further analyzed the impacts of factors such as climate, human activities, and land use on C_DOC_ across different river basins. In the Songhua River Basin, C_DOC_ exhibited a spatial pattern of “higher in the midstream and lower in the downstream”, a pattern driven by the combined effects of land use transition, anthropogenic emissions, and hydrological dilution. Existing studies indicate that intensive agricultural practices, specifically fertilization and irrigation, accelerate carbon leaching compared to stable natural ecosystems [51–53]. Additionally, urban wastewater effluents further elevate organic loads [54], whereas increased discharge downstream exerts a dilution effect [55]. These mechanisms are strongly corroborated by our SEM results (Fig. 10a), which reveal significant negative effects of land use (total effect: −0.34) and streamflow(total effect: −0.21) on C_DOC_, contrasted by a positive direct effect of human activities(total effect: 0.15). This statistical evidence confirms that the synergistic impact of agricultural and urban inputs in the midstream, coupled with hydrological dilution in the downstream, shapes the observed spatial heterogeneity. In the Yellow River Basin, C_DOC_ showed minimal variation across different reaches, a phenomenon that may be attributed to the counterbalancing effects of various influencing factors. In the Loess Plateau region of the midstream, soil and water conservation projects, such as terrace construction, check dam building, afforestation, and grassland restoration, effectively mitigated erosion and hence reduced the loss of dissolved organic carbon to river (erosive DOC) [56]; however, against the backdrop of global warming and decreased precipitation, river runoff decreased [57], which weakened the dilution capacity of the river and ultimately maintained C_DOC_ at a relatively stable level. In the Yangtze River Basin, C_DOC_ presented a spatial pattern of “significantly higher in the downstream than in the upstream and midstream”, and similarly, the Pearl River Basin exhibited a distinct increasing trend from the midstream to the downstream. These spatial variations were primarily driven by human activities in both the Yangtze River Basin (total effect: 0.38) and the Pearl River Basin (total effect: 0.68) (Fig. 10). The intense urbanization and agricultural development in the downstream regions of both basins generate continuous external organic matter inputs through agricultural runoff [58, 59], municipal wastewater [54], and industrial point-source pollution [60]. This is further supported by Spearman correlation analysis, which indicated that C_DOC_ was significantly positively correlated with anthropogenic factors such as GDP and population (Fig. 8a).

### Eco-hydrological coupling effects on temporal evolution of DOC

During 1997–2023, C_DOC_ and F_DOC_ increased across all basins at an annual mean rate of 0.04 mg L⁻¹ and 0.05 Tg yr⁻¹, respectively. These long-term trends reflected the dual influences of global warming and intensified human activities. The upward trend of C_DOC_, statistically significant in all basins (Fig. 3), stemmed from multiple interrelated factors. For example, human activities can alter land use types, and such alterations can, in turn, modify the nature and intensity of anthropogenic pressures, creating a feedback loop. Over the past three decades, China has undergone rapid economic development, and this economic development has made an important direct contribution to C_DOC_. Meanwhile, it also showed an indirect effect through nutrient enrichment caused by human activities, i.e., increased nutrient loadings promoted the production of autochthonous DOC in rivers [61, 62]. This study also confirmed that human activity factors have a significant positive correlation with C_DOC_, and the SEM results also revealed that human activities exert a certain positive effect on C_DOC_ (total effect: 0.34), suggesting that the observed C_DOC_ increase is likely attributable to intensified anthropogenic influences. Notably, in the past 30 years, China’s urbanization process has made rapid progress; the urbanization rate was only 26% in 1991, but it surpassed 67% by 2024. The Yangtze River Basin, the Yellow River Basin, and the Pearl River Basin in our study are the core areas of China’s urbanization process. In contrast, F_DOC_ changes were driven largely by streamflow across all the studied basins (total effect:0.78).

In terms of seasonal pattern, C_DOC_ exhibited a pronounced spring peak, a summer low, and a modest increase in autumn, as depicted in Fig. 4. The spring peak might be attributed to the release of DOC accumulated during winter, triggered by soil thawing during the snowmelt period [63, 64]. Additionally, root exudates produced in the period of early vegetation regrowth can also enhance soil DOC concentrations [65]. In summer, despite higher streamflow, C_DOC_ levels dropped to their annual minimum. This is because the elevated temperatures promoted microbial mineralization [66], driving hydrological dilution effects [67]. In autumn, moderate temperatures sustained microbial activity and slowed the DOC degradation, while reduced rainfall lessened dilution. Consequently, C_DOC_ remained at relatively elevated levels. Unlike C_DOC_, F_DOC_ displayed a clear seasonal pattern of elevated concentrations during the rainy season and reduced levels in the dry season (Fig. 7). This pattern was driven primarily by streamflow, particularly the increased flow rates during the summer flood season, which strengthened the physical mobilization of soil organic matter. Additionally, storm events can activate preferential flow pathways, rapidly transporting DOC from deeper soil layers to river channels [68, 69].

### Comparison of F_DOC_ between major Chinese rivers and global typical large rivers

We systematically compared the F_DOC_ of two major Chinese rivers (the Yangtze River and the Yellow River) with those of typical global large rivers (e.g., the Amazon River, Congo River, Mississippi River, Yenisei River, Red River, Yukon River, and Kolyma River). Our results show that the F_DOC_ of the Yangtze River (1.65 Tg yr^− 1^) is at a medium level globally, close to that of the Yukon River (1.47 Tg yr⁻¹) [70] and Kolyma River (1.78 Tg yr⁻¹) [70], slightly lower than the Red River (2.55 Tg yr⁻¹) [71], and significantly lower than the Mississippi River (4.28 Tg yr⁻¹) [72], Yenisei River (4.64 Tg yr⁻¹) [73], Congo River (8.9 Tg yr⁻¹) [72], and Amazon River (26.33 Tg yr⁻¹) [74]. In contrast, the F_DOC_ of the Yellow River (0.15 Tg yr⁻¹) is much lower than all the compared rivers. These F_DOC_ differences are jointly driven by natural factors such as runoff and climate (tropical rivers have abundant runoff and high vegetation carbon input [75], while Arctic rivers rely on permafrost thaw to supplement DOC [76]) and human disturbances (urbanization and agricultural activities in the lower reaches of the Yangtze River increase exogenous DOC input; soil and water conservation projects in the Yellow River reduce eroded DOC input, and its low runoff further restricts the flux). From a global perspective, the F_DOC_ characteristics of major Chinese rivers revealed in this study further improve the dataset on the regional diversity of global river F_DOC_. As the largest river in Asia, the medium F_DOC_ of the Yangtze River provides a key East Asian humid-region river case for global carbon cycle models. The extremely low F_DOC_ of the Yellow River offers a typical sample for understanding the carbon transport laws of rivers in arid and semi-arid regions, complementing those of the Amazon River (tropical humid region) and the Yenisei River (cold region). This highlights the regional representativeness and scientific value of Chinese rivers in global research on river carbon cycles across different climate zones and hydrological characteristics.

### Implications of the study findings for regional carbon management and global carbon cycle models

In light of the differences in spatiotemporal dynamics and driving mechanisms of DOC across the four major river basins, this study can provide differentiated strategies for regional carbon management: For the Songhua River Basin, focusing on the characteristic of relatively high C_DOC_ in the midstream dominated by croplands, precision fertilization can be implemented to control the excessive accumulation of soil organic carbon, and flood irrigation can be replaced with drip irrigation/spray irrigation to reduce DOC leaching, thereby regulating midstream C_DOC_ levels; for the Yellow River Basin, a balance between soil and water conservation and carbon export needs to be struck, and the stability of both C_DOC_ and F_DOC_ can be achieved by optimizing the coverage of forest and grass vegetation and reservoir operation schemes; for the Yangtze and Pearl River Basins, the focus should be on urbanization and exogenous DOC input from agricultural activities, with DOC controlled through wastewater treatment and the construction of ecological ditches, and the seasonal balance of F_DOC_ maintained by regulating streamflow via reservoirs. Additionally, the long-term DOC data (1997–2023) from this study can serve as a quantitative benchmark for evaluating management effectiveness, facilitating the establishment of a “monitoring-evaluation-optimization” closed-loop system.

Furthermore, the 1922 sets of measured C_DOC_ data and derived F_DOC_ data collected in this study can provide key support for global carbon cycle models. This dataset lays a data foundation for the long-term research on DOC in typical basins of China, serving as a key resource for model calibration. The driver relationships quantified by Structural Equation Modeling (SEM), such as the total effects of streamflow on F_DOC_ (0.83) and climatic factors on C_DOC_ (−0.22), also offer a basis for revising critical parameters in these models, which ultimately enhances the simulation accuracy for DOC dynamics in Chinese rivers.

### Uncertainties and limitations

This study is constrained by the spatiotemporal coverage of the data. Although the dataset covers multiple climatic zones and land uses, the sample sizes for the Songhua and Pearl River basins are insufficient. Consequently, we can only estimate the F_DOC_ into the sea for these two major rivers (the Yangtze River and the Yellow River), lacking estimates for other rivers. Our study also assumes that population and economic growth are positively correlated with pollutant emissions, but data availability constraints limit further analysis of the underlying driving mechanisms. Furthermore, the regulatory effects of hydrological engineering and other human activities on riverine DOC transport remain poorly understood. In addition, our understanding of how hydraulic infrastructure (e.g., dams) regulates riverine organic carbon transport is still incomplete. Dams are widespread globally and can significantly alter riverine DOC transport by increasing water residence time and promoting in-reservoir sedimentation [77]. However, in this study, we only accounted for the number of hydraulic structures in a crude manner, without fully considering their complex impacts on carbon cycling. Moreover, the river discharge data used here are based on the GloFAS-ERA5 reanalysis product. Although this dataset is skillful in 86% of catchments [78], it nonetheless carries inherent uncertainties. We also conducted spatial autocorrelation tests and found evidence of spatial autocorrelation in our dataset. This phenomenon may influence the apparent relationships among variables, potentially leading to an overestimation of the contributions of certain factors in the SEM analysis. However, the SEM framework employed in this study cannot directly account for spatial autocorrelation. We note that several previous studies also did not explicitly incorporate spatial autocorrelation [79, 80]. Therefore, while the SEM results provide useful insights into the drivers of riverine DOC, readers should interpret the findings with caution, considering that spatial autocorrelation may introduce additional uncertainties. Future research should comprehensively consider the impact of hydrological engineering projects on DOC transport, account for spatial autocorrelation in the data, and improve the estimation of F_DOC_ for all of China’s rivers flowing into the ocean by integrating multi-source datasets.

## Conclusion

This study systematically analyzed the spatiotemporal variations and driving mechanisms of DOC across China’s four major river basins—the Songhua River Basin, Yellow River Basin, Yangtze River Basin, and Pearl River Basin—during 1997–2023. Spatially, C_DOC_ displayed a latitudinal “north high, south low” gradient, with the highest concentration (7.85 ± 1.01 mg L⁻¹) observed in the Songhua River Basin, attributed to permafrost thawing and soil organic carbon release in northern black soil regions. In contrast, southern basins showed lower C_DOC_ values but higher F_DOC_ values owing to elevated streamflow. Generally, C_DOC_ and F_DOC_ both exhibited diverse longitudinal variations across the studied basins. Temporally, C_DOC_ and F_DOC_ both exhibited statistically significant upward trends (0.04 mg L⁻¹ yr⁻¹ and 0.05 Tg yr⁻¹, respectively), driven by anthropogenic emissions and streamflow dynamics. Seasonally, C_DOC_ showed a “spring peak, summer trough” pattern because of snowmelt, microbial activity, and dilution effects, while F_DOC_ aligned with streamflow, peaking in summer. Overall, SEM showed that climate and anthropogenic factors operate as dual regulators of C_DOC_, while F_DOC_ was regulated predominantly by streamflow (total effect: 0.83). The results of this study provide critical evidence for understanding the coupled natural–anthropogenic mechanisms of carbon cycling in China’s large river basins, laying a foundation for regional carbon management and refining global carbon models.

## Supplementary Information

Supplementary Material 1

## Data Availability

The CDOC data collected from the literature have been uploaded to 10.5281/zenodo.15293125 and are available for public download. The land use data were obtained from the 30-m Chinese Land Cover Dataset (1985–2023) published by the Yang Jie and Huang Xin team at Wuhan University (https://zenodo.org/records/12779975). The streamflow data were obtained from the River discharge and related historical data from the Global Flood Awareness System (v 4.0) (10.24381/cds.a4fdd6b9). Data for two climatic factors were obtained from separate datasets: precipitation data from the 1-km monthly precipitation dataset for China (1901–2024) (10.5281/zenodo.3114194), and air temperature data from the 1-km monthly mean temperature dataset for China (1901–2024) (10.11888/Meteoro.tpdc.270961) The population density data were obtained from the Chinese Population Spatial Distribution Kilometer Grid Dataset (https://www.resdc.cn/DOI/DOI.aspx?DOIID=32). The GDP data were extracted from the Chinese GDP Spatial Distribution Kilometer Grid Dataset (https://www.resdc.cn/DOI/DOI.aspx?DOIID=33). The hydroelectric power station and reservoir data were derived from a global dataset combining open-source hydropower plant and reservoir data (10.1038/s41597-025-04975-0). The soil organic carbon data were obtained from a China dataset of soil properties for land surface modelling (version 2, CSDLv2) (10.5194/essd-17-517-2025). The river network data were derived from the GRADES dataset (Global Reconstruction of Naturalized River Discharge at 2.94 Million River Reaches) (10.11888/Terre.tpdc.272898).
