# Supplementary material for: Spatiotemporal variations in dissolved organic carbon in China’s major river basins and their associations with climate change and human activities
Source: Carbon Balance Manag. 2025 Dec 27;21:25. doi: 10.1186/s13021-025-00387-0 (PMC12853768; doi:10.1186/s13021-025-00387-0)
Supplement: Supplementary file 1 — Supplementary Material 1 [file 13021_2025_387_MOESM1_ESM.docx]

**Table S1** Land use type proportions in the four major river basins (Songhua River Basin, Yellow River Basin, Yangtze River Basin, and Pearl River Basin).

| River Basin | Cropland (%) | Forest (%) | Shrub (%) | Grassland (%) | Water (%) | Snow/Ice (%) | Barren (%) | Impervious (%) | Wetland (%) |
| --- | --- | --- | --- | --- | --- | --- | --- | --- | --- |
| Songhua River | 45.26±0.73 | 42.86±0.41 | / | 7.00±0.69 | 1.27±0.12 | / | 0.53±0.10 | 3.05±0.54 | 0.03±0.01 |
| Yellow River | 23.98±0.93 | 10.70±0.68 | 0.56±0.08 | 58.5±0.60 | 0.67±0.06 | 0.05±0.01 | 3.37±0.53 | 2.13±0.49 | 0.04±0.02 |
| Yangtze River | 29.53±0.62 | 46.24±0.47 | 0.64±0.09 | 18.79±0.38 | 2.03±0.09 | 0.17±0.02 | 0.82±0.10 | 1.78±0.53 | / |
| Pearl River | 27.14±0.63 | 65.99±0.64 | 2.25±0.30 | 1.01±0.22 | 1.60±0.12 | / | 0.01±0.00 | 2.00±0.54 | / |

**Table S2** Detailed quantitative characteristics of each of the four basins (Songhua River Basin, Yellow River Basin, Yangtze River Basin, and Pearl River Basin).

| River Basin | Area(km^2^) | Streamflow (Bm^3^) | Precipitation (mm) | Air Temperature (℃) | Soil Organic Carbon (g/kg) | Population (persons/km^2^) | GDP (10,000 yuan/km²) | Number of Hydroelectric Power Station and Reservoir |
| --- | --- | --- | --- | --- | --- | --- | --- | --- |
| Songhua River | 580887 | 1426.8±425.7 | 539.1±73.6 | 3.0±0.5 | 412.4±213.7 | 95±6 | 208.7±146.6 | 8±2 |
| Yellow River | 797955 | 576.2±128.7 | 473.8±46.5 | 6.6±0.4 | 247.4±256.0 | 141±11 | 402.3±359.3 | 61±32 |
| Yangtze River | 1800027 | 9889.6±1254.6 | 1042.9±71.2 | 11.6±0.3 | 343.9±256.0 | 246±15 | 733.6±759.8 | 287±194 |
| Pearl River | 543446 | 4693.1±1001.3 | 1495.8±154.4 | 19.8±0.4 | 256.9±392.7 | 295±50 | 1003.5±964.4 | 53±29 |

**Table S3** Changes in Land Use Composition of Major River Basins in China (Songhua River Basin, Yellow River Basin, Yangtze River Basin, and Pearl River Basin), 1996–2023 (in Percentage Points)

| River Basin | Cropland | Forest | Shrub | Grassland | Water | Snow/Ice | Barren | Impervious | Wetland |
| --- | --- | --- | --- | --- | --- | --- | --- | --- | --- |
| Songhua River | 0.28 | -1.61 | 0.01 | -0.32 | 0.22 | 0.00 | -0.28 | 1.73 | -0.03 |
| Yellow River | -1.91 | 2.11 | -0.18 | 0.01 | 0.12 | 0.01 | -1.61 | 1.52 | -0.06 |
| Yangtze River | -1.94 | 1.63 | -0.26 | -1.24 | -0.04 | -0.03 | 0.23 | 1.64 | 0.00 |
| Pearl River | -0.70 | 0.86 | -1.12 | -0.66 | -0.14 | 0.00 | 0.00 | 1.76 | 0.00 |
